# Functional Connectivity Within the Gustatory Network Is Altered by Fat Content and Oral Fat Sensitivity – A Pilot Study

**DOI:** 10.3389/fnins.2019.00725

**Published:** 2019-07-10

**Authors:** Sabine Frank-Podlech, Jaana M. Heinze, Jürgen Machann, Klaus Scheffler, Guido Camps, Andreas Fritsche, Melanie Rosenberger, Jörg Hinrichs, Ralf Veit, Hubert Preissl

**Affiliations:** ^1^Institute for Medical Psychology and Behavioural Neurobiology, University of Tübingen, Tübingen, Germany; ^2^Institute for Diabetes Research and Metabolic Diseases of the Helmholtz Center Munich at the University of Tübingen, German Center for Diabetes Research (DZD), Tübingen, Germany; ^3^Department of Internal Medicine IV, University Hospital, Tübingen, Germany; ^4^Section on Experimental Radiology, Department of Diagnostic and Interventional Radiology, University Hospital, Tübingen, Germany; ^5^Department of Biomedical Magnetic Resonance, University of Tübingen, Tübingen, Germany; ^6^Department of High-Field Magnetic Resonance, Max Planck Institute for Biological Cybernetics, Tübingen, Germany; ^7^Division of Human Nutrition, Wageningen University & Research, Wageningen, Netherlands; ^8^Department Soft Matter Science and Dairy Technology, University of Hohenheim, Stuttgart, Germany; ^9^Institute for Diabetes and Obesity, Helmholtz Diabetes Center, Helmholtz Center Munich, German Research Center for Environmental Health, Neuherberg, Germany; ^10^Department of Pharmacy and Biochemistry, Faculty of Science, University of Tübingen, Tübingen, Germany

**Keywords:** functional connectivity, hypothalamus, striatum, nucleus tractus solitarii, oral fat sensitivity, fat detection threshold, nutritional fat

## Abstract

**Background:** The amount of fat in ingested food dictates specific activation patterns in the brain, particularly in homeostatic and reward-related areas. Taste-specific brain activation changes have also been shown and the sensitivity to the oral perception of fat is associated with differential eating behavior and physiological parameters. The association between oral fat sensitivity and neuronal network functions has, however, not yet been defined.

**Objective:** We aimed to investigate the association between fat-dependent neuronal functional connectivity patterns and oral fat sensitivity.

**Design:** To investigate the underlying changes in network dynamics caused by fat intake, we measured resting-state functional connectivity in 11 normal-weight male participants before and after a high- vs. a low-fat meal on two separate study days. Oral fat sensitivity was also measured on both days. We used a high-resolution functional magnetic resonance imaging (MRI) sequence to measure any connectivity changes in networks with the seed in the brainstem (nucleus tractus solitarii, NTS), in homeostatic (hypothalamus) and in reward regions (ventral and dorsal striatum). Seed-based functional connectivity (FC) maps were analyzed using factorial analyses and correlation analyses with oral fat sensitivity were also performed.

**Results:** Regardless of fat content, FC between NTS and reward and gustatory areas was lower after ingestion. Oral fat sensitivity was positively correlated with FC between homeostatic regions and limbic areas in the high-fat condition, but negatively correlated with FC between the dorsal striatum and somatosensory regions in the low-fat condition.

**Conclusion:** Our results show the interaction of oral fat sensitivity with the network based neuronal processing of high- vs. low-fat meals. Variations in neuronal connectivity network patterns might therefore be a possible moderator of the association of oral fat sensitivity and eating behavior.

## Introduction

Certain brain areas are highly responsive to the intake of high-caloric meals. However, generally speaking, information about the ingested nutrients is on the central level firstly processed in the brainstem, particularly in the NTS, a major afferent target of the vagus nerve, and in the diencephalon (in the hypothalamus), the major homeostatic region in the brain (Morton et al., 2014; Neseliler et al., 2017). Animal studies have shown that the NTS is deeply involved in the coding of taste qualities (Di Lorenzo and Victor, 2003) and shows a direct connection to the hypothalamus which is also relevant for processes of fat intake (DiPatrizio and Piomelli, 2015). While the NTS is rarely investigated in human functional imaging studies due to technical limitations and restricted Field of View (FOV), the influence of food intake on the hypothalamus has been described more frequently.

Thus, it was shown that the hypothalamic activation is markedly lower after sugar intake or after a high-fat meal than after the intake of water or of a low-fat meal (Smeets et al., 2005a,b; Frank et al., 2012; Eldeghaidy et al., 2016). Homeostatic processes including hunger and satiety are mainly processed in predesignated nuclei within the hypothalamus (Morton et al., 2014). However, the human brain imaging methods currently available cannot resolve these small structures. Due to this limitation, several groups proposed a separation scheme of the hypothalamus to investigate specific regional effects in the human hypothalamus. Smeets et al. (2005b) divided the hypothalamus into the upper anterior, upper posterior, lower anterior and lower posterior hypothalamus. The most pronounced effect of glucose ingestion was observed in the upper anterior hypothalamus (Smeets et al., 2005a,b). Using specific coordinates in the lateral and in the ventromedial part of the hypothalamus, Kullmann et al. (2014) investigated resting state FC on the basis of detailed human hypothalamic anatomical investigations. These two seeds revealed two distinguishable networks which were particularly well connected to dopaminergic reward regions such as the dorsal and ventral striatum (VS).

The VS was shown to be sensitive to the motivational relevance of a stimulus and associated with goal-directed behavior, whereas the DS codes for a more complex signal that incorporates reward probability and tends to be associated with habitual behavior (Belin and Everitt, 2008; Tricomi and Lempert, 2015). In accordance with this, resting-state FC patterns of the dorsal and the VS to cortical regions were observed to be body-mass-index (BMI) dependent (e.g., higher connectivity between the DS and the somatosensory cortex with increased BMI) (Contreras-Rodriguez et al., 2017).

Brain connectivity patterns might also be influenced by gastric emptying, which differs according to the fat content in food, with slower gastric emptying after ingestion of a high-fat meal (Luscombe-Marsh et al., 2013). Viscosity differences play a further, crucial role for gastric emptying, with higher viscosity being associated with slower gastric emptying (Marciani et al., 2001; Camps et al., 2016). Thus, when investigating neuronal connectivity patterns associated with fat ingestion, it is appropriate to account for peripheral gastric emptying patterns.

Besides peripheral and central processes, ingested food is first perceived in the oral cavity. In the last few years, the topic of oral fat sensitivity has attracted more attention (Mattes, 2010; Keast and Constanzo, 2015). Several studies have shown that oral fat perception – and thus fat sensitivity – can be measured using free fatty acids (Mattes, 2005, 2009; Newman and Keast, 2013; Haryono et al., 2014; Heinze et al., 2017). Fat sensitivity can be affected by several factors such as BMI and dietary fat intake. Stewart et al. (2010) showed that increased fat sensitivity was associated with lower BMI as well as with lower energy and fat intake. Subjects who were hyposensitive to oleic acids consumed more energy, fat, saturated fat, and fatty foods, and had a higher BMI than hypersensitive subjects (Stewart et al., 2011). Beta carotene consumption (as found in many fruits and vegetables) was negatively associated with detection thresholds (and thus positively with fat sensitivity) for various fatty stimuli (oleic acid, paraffin oil, and canola oil spiked with oleic acid). However, a single high-fat meal prior to fat taste threshold testing did not affect oral fat sensitivity (Newman et al., 2016). In addition, high oral sensitivity to canola oil was seen to be associated with high intake of high-fat foods (Heinze et al., 2018).

With regard to brain functions, drops of fatty solutions on the tongue are processed in different networks including homeostatic, reward or gustatory areas depending on the pleasantness, fattiness or texture of the stimuli (Grabenhorst et al., 2010).

However, until now it was unclear which neuronal processes are related to the sensitivity of oral fat perception (Heinze et al., 2015). In the current study, we therefore investigated both oral fat sensitivity and FC changes due to a high- vs. a low-fat meal. We hypothesized changing connectivity network patterns with the seed in hypothalamic, striatal and NTS regions after food intake, dependent on the fat content of a meal. In addition, we assumed differential connectivity patterns dependent on individual oral fat sensitivity.

## Materials and Methods

### Participants

We screened 15 healthy, normal-weight men, 4 of who were unable to participate due to age (*n* = 1), scheduling difficulties (*n* = 2), or claustrophobia (*n* = 1). The remaining 11 men participated in the study (BMI: 23.1 kg/m^2^ ± 2.0 SD, age: 24.6 years ± 2.4 SD). Eligibility criteria consisted of self-reported unimpaired taste function, no chronic or acute diagnosed diseases, and no allergies to dairy products. Furthermore, only participants with no contraindications for Magnetic Resonance Imaging (MRI) measurements (such as metal implants) were recruited. All participants gave written informed consent prior to participation and the study was approved by the Ethics Committee of the University of Tübingen. The study was conducted in accordance with The Code of Ethics of the World Medical Association (Declaration of Helsinki) for experiments involving humans. None of the participants suffered from any kind of disturbed eating behavior or psychiatric abnormality, as assessed by the Three Factor Eating Questionnaire (THEQ) (Pudel and Westenhöfer, 1989), Eating Disorder Examination (EDE) (Hilbert et al., 2004), and the Patient Health Questionnaire (PHQ) (Löwe et al., 2002). Prior to and after the scanning session and before the sensory testing, participants were requested to rate their current state of hunger, appetite, satiety and feeling of fullness on a 0–100 visual analog scale (VAS, 0 = not at all, 100 = extremely) on both measurement days. Height and weight were assessed during the first study appointment and BMI was calculated.

### Study Design (Figure 1)

Following an overnight fast, each participant came to the imaging facilities twice and gave written informed consent at the beginning of the first measurement day. Having completed the VAS for the first time, the participant was placed in a supine position in the scanner and both gastric content and resting-state functional brain activity were measured in the fasted state. The scanner table was later moved out of the scanner and, while ingesting 500 ml of yogurt within 10 min in a sitting position, the participant rated the yogurt with regard to its pleasantness and sensory qualities (creamy, fatty, sweet, and sour). On one of the two measurement days, the yogurt contained 8% fat (high-fat condition), on the other day <0.01% fat (low-fat condition). The order of the conditions was counterbalanced. After the 10 min period of yogurt ingestion and rating, the participant was again moved in the scanner and the gastric content was measured once more to determine gastric filling. Resting-state brain activity was again measured 40 min after commencing yogurt ingestion. Gastric content was then measured a third time to assess gastric emptying. Following functional MRI (fMRI) measurements, participants filled in the VAS again as well as further questionnaires. Finally, the VAS was filled in a third time before the detection threshold (= oral fat sensitivity) for oleic acid was determined.

**FIGURE 1:**
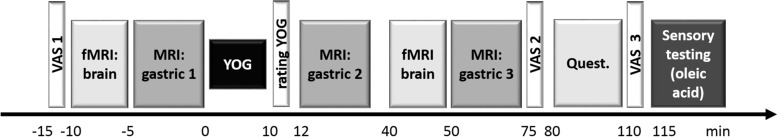
Study design: VAS, visual analog scale; YOG, yogurt.

### Imaging Methods

Scanning was performed on a 3-T whole-body scanner (MAGNETOM Prisma, Siemens Healthineers, Erlangen, Germany) with a standard 20-channel head coil for brain measurements and a combination of 32-channel spine-array and 18-channel body-array coil for gastric measurements.

For resting-state functional imaging, an echo-planar imaging sequence with a reduced FOV was used to investigate brainstem and subcortical regions as well as striatum, insula, operculum, and temporal region with an improved signal-to-noise ratio and decreased distortion (Olman et al., 2009; Kullmann et al., 2018) (TR = 3 s, TE = 34 ms, echo-spacing 0.66 ms, FOV = 192 × 64 × 90 mm^3^, matrix 96 × 32 × 36, refocusing flip angle 90°, voxel size 2 × 2 × 2 mm^3^, slice thickness 2 mm, 0.5 mm gap and the images were acquired in interleaved order). Each brain volume comprised 36 transversal slices and each functional run contained 60 image volumes (3 min) (Kullmann et al., 2018). All participants were instructed not to focus their thoughts on anything in particular and to keep their eyes closed during the resting-state MR acquisition.

In addition, high-resolution T1 weighted anatomical images (MP-RAGE: 176 slices, matrix: 256 × 224, 1 × 1 × 1 mm^3^) of the brain were obtained.

Gastric volume was measured using a method similar to the one previously described (Camps et al., 2016) with a T2-weighted spin-echo sequence (TR = 800 ms, TE = 83 ms, FOV = 296 × 380 mm^2^, matrix 250 × 320, refocusing flip angle 107°, voxel size 1.19 × 1.19 × 7.5 mm^3^, slice thickness 5 mm, 2.5 mm gap, TA 19 s in breath-hold and the images were acquired in interleaved order). During the measurement, the participants were instructed to hold their breath on expiration so that the position of the diaphragm and stomach could be fixated.

### Yogurt Production

The low- and high-fat yogurt used in our study was produced at the Institute of Food Science and Biotechnology (University of Hohenheim) (see Frank et al. (2012) so as to provide two yogurt meals with comparable viscosity but with different fat content (for more details, see Supplementary Table S1).

### Yogurt Rating

During yogurt ingestion, participants rated the product for its creaminess, fattiness, sweet, sour and palatability on a VAS.

### Oral Sensitivity Testing

The determination of detection threshold for oleic acid was based on the ASTM E679 method (American Society For Testing And Materials [ASTM], 2011), ISO 3972:2011 – Sensory analysis –Methodology –Method of investigating sensitivity of taste (International Standard Organisation [ISO], 2011), and the protocols of Haryono et al. (2014) and Giguere et al. (2016) (for details see Supplementary Table S2).

Using standard sensory testing methods, a triangle forced-choice test was applied to determine the detection threshold. Here, participants received 8 rows, each with a sets of 3 samples (one spiked with oleic acid) in ascending order, with the bottom row containing the lowest concentration. After tasting all 3 samples of a set, the participants were asked to identify the “odd one out.” To avoid olfactory and visual cues, participants were instructed to wear nose clips and red glasses during testing. For more information see Supplementary Material. We determined an individual fat sensitivity index (inverse detection threshold) for each participant. These ranged from 1 (non-taster) to 9 (detection of the lowest concentration).

### Analyses

#### Preprocessing of Imaging Data

Preprocessing of brain fMRI resting-state data was performed using Data Processing Assistant for Resting-State fMRI^1^ (Chao-Gan and Yu-Feng, 2010) which is based on statistical parametric mapping (SPM12)^2^ and Resting-State fMRI Data Analysis Toolkit^3^ (Song et al., 2011). Functional images were realigned and co-registered to the anatomical image. The structural image was normalized to the Montreal Neurological Institute template using DARTEL, and the resulting parameter file was used to normalize the functional images. Normalized images were smoothed with a three-dimensional isotropic Gaussian kernel (full-width at half-maximum (FWHM): 4 mm). A temporal filter (0.01–0.08 Hz) was applied to reduce low frequency drifts and high frequency physiological noise. Nuisance regression was performed using white matter, cerebrospinal fluid (CSF), and the six head motion parameters as covariates. None of the participants displayed head motion with more than 0.5 mm maximum displacement or 0.5° of any angular motion.

#### Seed Based Functional Connectivity

Functional connectivity maps of resting-state measurements were obtained using seed- based analyses by computing FC between preselected seeds and each voxel within the FOV. To investigate the connectivity patterns associated with food intake, five seeds were defined as follows: the NTS, as an early taste-sensitive region in the brainstem (Hill and May, 2007), was defined by the mask based on coordinates by Hoogeveen et al. (2015) with a sphere of 3 mm. On the basis of an earlier connectivity study (Kullmann et al., 2014), masks (sphere 3 mm) of the lateral (LH) and the ventromedial part of the hypothalamus (VMH) were chosen as further seeds (Kullmann et al., 2014). To investigate connectivity pattern of food reward related regions, masks of the dorsal (DS) and VS were built anatomically in accordance with Mawlawi et al. (2001) since these regions are larger and structurally distributed over a wider area.

#### Main Effects of Connectivity Changes

Using SPM12, an independent whole field of view FC analysis was performed for each of the described five seed regions. For each analysis, baseline-corrected FC maps (post-pre measurement) were included in a full-factorial model with the covariates “subjective fattiness rating” of the yogurt, “gastric emptying” (gastric 3 - gastric 2) and “hunger change” (VAS 2-VAS 1). Brain regions exceeding a threshold of *p* < 0.05 family wise error (FWE) corrected for multiple comparisons at cluster level after an initial threshold of *p* < 0.001 uncorrected with an extended voxel threshold of 5 voxels were considered significant. Results are reported with and without Bonferroni correction for the number of seed-based analyses (one analysis for each of the 5 seeds: *p*_*Bonferroni*_ = 0.01).

#### Preprocessing of Gastric Emptying Data

Gastric volume was quantified applying an in-house developed software using Matlab (Matlab^®^, The MathWorks Inc., version R2014a). At each time point (gastric 1, gastric 2, gastric 3), gastric volume was calculated by multiplying the surface area of gastric content per slice with the slice thickness, including the gap distance, and summed over the total slices to estimate gastric content.

#### Behavioral Analyses and Statistical Analyses of Gastric Emptying Data

Analyses were performed using SPSS, version 24.0 (IBM^®^ SPSS^®^, Armonk, NY, United States).

Differences between high- and low-fat yogurt ratings were analyzed using paired *t*-tests.

A repeated 2 × 2 measurement ANOVA with baseline-corrected data was conducted to test time- and yogurt-dependent effects within hunger parameters.

Differences in oral fat sensitivity due to the earlier ingestion of a high- vs. low-fat meal were investigated using paired *t*-test. For test-retest reliability, we performed correlation analyses of oral fat sensitivity during the two study days.

Differences in pre-measurement gastric volume were analyzed using a paired *t*-test to ensure that conditions were identical on both measurement days with regard to gastric volume. Changes in gastric volume were analyzed by a 3 × 2 (time x yogurt) ANOVA. In the event of significant time × yogurt interactions, Bonferroni corrected *post hoc* paired *t*-tests were performed. To evaluate associations between variables, Pearson correlations were performed.

A *p*-value of *p* < 0.05 was considered significant for all behavioral analyses. Data are presented as mean ± SD.

#### Regression Analyses of Neuronal Connectivity Patterns With Oral Fat Sensitivity

To investigate the association of changes in FC with the individual oral fat sensitivity, additional whole FOV regression analyses of resting-state connectivity changes were performed in SPM in each seed region for the high-fat and the low-fat day separately. Any brain regions exceeding a threshold of *p* < 0.05 FWE corrected for multiple comparisons at cluster level after an initial threshold of *p* < 0.001 uncorrected with an extended voxel threshold of 5 voxels were considered significant. Results are reported with and without Bonferroni correction for the number of seed-based analyses (two analyses for each of the five seeds: *p*_*Bonferroni*_ = 0.005). Differences between resulting correlations of changes in FC with oral fat sensitivity for both study days were calculated by transformation of the correlation coefficients to Fisher’s *Z* values which were then analyzed for significant differences using the online platform psychometrica^4^.

#### Regression Analyses of Neuronal Connectivity Patterns With Gastric Volume

Associations between gastric emptying and FC changes were investigated at an explorative level by regression analyses. As was the case in regression analyses of connectivity patterns with oral fat sensitivity, whole FOV regression analyses of resting-state connectivity changes with gastric emptying were performed in SPM with each seed region for the high-fat and the low-fat day separately. Brain regions were considered significant if they exceeded a threshold of *p* < 0.05 FWE corrected for multiple comparisons at cluster level after an initial threshold of *p* < 0.001 uncorrected with an extended voxel threshold of 5 voxels.

## Results

### Behavioral Data

No significant differences were observed for the different yogurt types with regard to hunger, satiety, appetite, and feeling of fullness.

### Yogurt Rating

The rating of the yogurt during ingestion showed significantly higher creaminess (*t*_10_ = 3.49, *p* = 0.006, η^2^ = 0.55), fattiness (*t*_10_ = 3.10, *p* = 0.011, η^2^ = 0.49) and palatability (*t*_10_ = 4.73, *p* = 0.001, η^2^ = 0.70) ratings for the high-fat yogurt. Both, creaminess and palatability ratings correlated significantly with fattiness (*r* = 0.734; *p* < 0.001 and *r* = 0.439, *p* < 0.05).

### Gastric Volume

Pre-measurement gastric volume did not differ significantly between the participants. However, in the high-fat yogurt condition, participants had a different gastric volume and emptying, indicated by higher gastric volume to both post measurements as shown by a significant ANOVA main effect of yogurt (*F*_10,20_ = 30.43, *p* < 0.001, η^2^ = 0.75), time (*F*_10,20_ = 501.06, *p* < 0.001, η^2^ = 0.98) and a significant time × yogurt interaction (*F*_10,20_ = 16.26, *p* < 0.001, η^2^ = 0.62). Bonferroni corrected *post hoc* paired *t*-tests differed significantly 10 min after ingestion start [gastric 2: (t_10_ = 3.69, *p* = 0.004)] and still did so after 50 min [gastric 3: (t_10_ = 6.45, *p* < 0.001)]. What is more, gastric emptying differed between the yogurt conditions, indicated by *post hoc* tests showing a lower reduction in gastric volume from the gastric 2 to gastric 3 time point in the high-fat condition (t_10_ = 0.017, *p* < 0.001) (Supplementary Figure S1). Gastric emptying was not significantly correlated either to hunger ratings or to the palatability of the yogurt conditions.

### Oral Sensory Perception

Paired *t*-tests showed no significant difference between the yogurt conditions for oral fat sensitivity (high-fat day: 3.8 ± 1.47 SD; low fat day: 3.8 ± 1.60 SD). Fat sensitivity was positively correlated on both days (*r* = 0.664, *p* = 0.026).

### Functional Connectivity

#### Main Effects of Connectivity Changes

**FIGURE 2:**
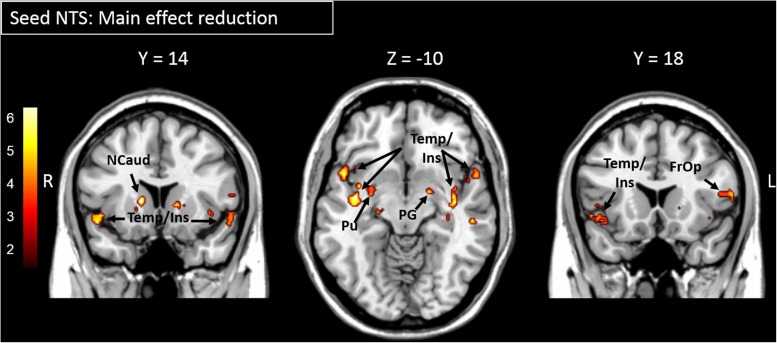
Areas showing significantly reduced functional connectivity to the NTS after yogurt intake are presented. Color bar depicts *z*-values. FrOp, Frontal Operculum; Ins, Insula; Ncaud, Nucleus caudatus; NTS, Nucleus Tractus Solitarii; PG, Globus Pallidus; Pu, Putamen; Temp, temporal cortex.

The main effect of yogurt intake showed reduced FC between the NTS and the putamen (part of the DS), the globus pallidus and the caudate (part of the VS) as well as regions within the insular and the temporal cortex (Figure 2 and Table 1).

**TABLE 1:** Significant effects of changes in functional connectivity patterns due to meal ingestion (high- vs. low-fat yogurt meal) based on full factorial analyses and regression analyses.

**Analyses**	**Seed**	**Brain Region**	**MNI Coordinates**			**Cluster size (in voxels)^§^**	***Z* value**	***p* FWE_cluster_**
			***x***	***y***	***z***			
**Full factorial analyses**								
Reduction	NTS	DS (peak in Caudate)	−14	14	6	28	5.05	0.025
		Temporal cortex/ Insula	52	−26	−6	41	4.63	0.003^*^
			40	−8	−12	84	4.38	<0.001^*^
			56	10	−6	99	4.14	<0.001^*^
		Temporal cortex/ Insula	−40	−6	−10	91	4.56	<0.001^*^
			−48	12	−10	136	4.47	0.007^*^
		Globus pallidus	18	−2	−10	33	4.01	0.010
		Frontal Operculum	60	18	10	34	3.91	0.009^*^
		DS (peak in Putamen)	−28	−4	−10	27	3.67	0.030
**Regression analyses with oral fat sensitivity within the high-fat condition**								
Positive correlation	VMH	Limbic lobe with Hippocampus and Amygdala	16	4	−16	84	5.03	<0.001^*^
		Temporal cortex	−56	−24	−8	55	4.42	<0.001^*^
			−52	−16	−12	52	4.25	<0.001^*^
		Supra Marginal	−50	−26	28	17	4.21	0.016
		Temporal cortex	56	8	−5	45	3.89	<0.001^*^
**Regression analyses with oral fat sensitivity within the low-fat condition**								
Negative correlation	DS	Somatosensory cortex	−58	−16	14	14	3.73	0.048

^*^ Bonferroni corrected significant for the number of separate analyses. Full factorial design (5 analyses: *p* = 0.05/5 = 0.01), Regression analyses (10 analyses: *p* = 0.05/10 = 0.005). ^§^ The cluster size of FWE (family-wise error) corrected clusters, based on a primary uncorrected threshold level of *p* < 0.001. DS, dorsal striatum; MNI, Montreal Neurological Institute; NTS, Nucleus Tractus Solitarii; ROI, region of interest; VMH, ventromedial hypothalamus.

No main effect of connectivity changes due to yogurt intake was found for the other seed regions on the basis of the significant threshold used. No significant differential effect for the high- vs. low-fat condition was observed.

#### Regression Analyses With Oral Fat Sensitivity

Regression analyses with oral fat sensitivity revealed significant correlations in the analyses with the seeds VMH and DS.

**FIGURE 3:**
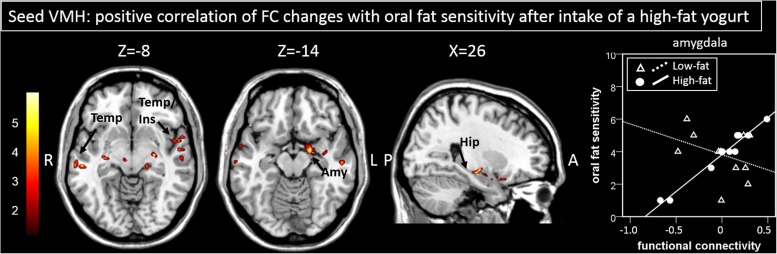
Areas showing a significant positive correlation of changes in functional connectivity to the seed region (here VMH) are presented. The correlation of functional connectivity changes between the VMH and the amygdala with oral fat sensitivity for the high-fat condition (significant positive correlation) and for the low-fat condition (not significant, by way of comparison) as shown by the scatterplot is exemplary. Color bar depicts *z*-values. Amy, Amygdala; FC, functional connectivity; Hip, hippocampus; Ins, Insula; Temp, temporal cortex, VMH, ventromedial hypothalamus.

Following ingestion of the high-fat yogurt, regression analysis with oral fat sensitivity revealed stronger connectivity changes of the VMH to regions within the limbic lobe including hippocampal and amygdalar areas as well as to temporal regions (Figure 3 and Table 1, correlation of oral fat sensitivity exemplarily depicted for amygdalar activity at *x* = 20, *y* = −2, *z* = −14: low-fat *r* = −0.36, *p* = 0.28; high-fat *r* = 0.983, *p* < 0.001; the two correlations are significantly different with *z* = 5.51, *p* < 0.001).

**FIGURE 4:**
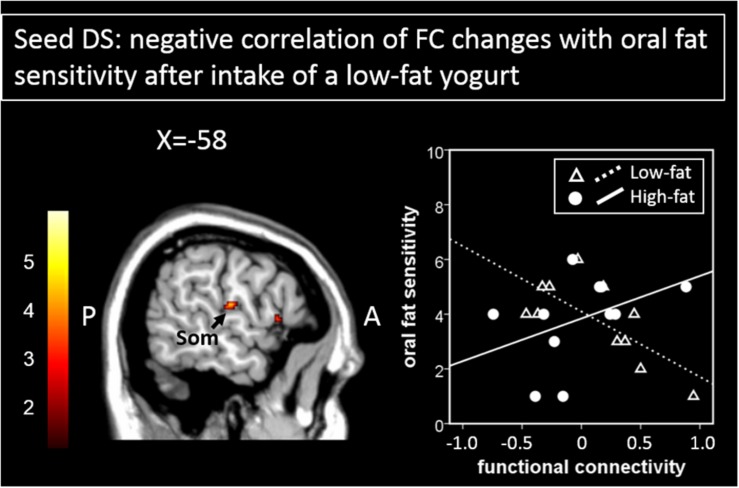
Areas showing a significant negative correlation of functional connectivity changes to the seed region (here DS) with oral fat sensitivity are presented here. The scatterplot shows the correlation of functional connectivity patterns between the DS and the somatosensory cortex with oral fat sensitivity for the low-fat condition (significant negative correlation) and for the high-fat condition (not significant, by way of comparison). Color bar depicts *z*-values. DS, dorsal striatum; Som, somatosensory cortex.

Furthermore, when using the DS as a seed region, regression analyses between oral fat sensitivity and functional connectivity changes showed significant negative correlations for the FC changes between the DS and the somatosensory cortex in the low-fat condition (*r* = −0.72, *p* = 0.01) but not for the high-fat condition (*r* = 0.42, *p* = 0.20, Figure 4 and Table 1). The two correlations differ significantly with *z* = 2.73, *p* = 0.003.

No effects were observed for FC changes and gastric emptying.

## Discussion

Our results reveal distinct altered connectivity patterns following the intake of a high- and a low-fat meal as well as differential connectivity when taking oral fat sensitivity into account. Thus, this study constitutes a first step toward overcoming the knowledge gap about the interaction of oral fat sensitivity and neuronal functions as claimed in Heinze et al. (2015).

Imaging data of the present study revealed that the NTS, as an early stage taste processing region, shows decreased FC to reward and gustatory areas following the ingestion of yogurt. In general, at the central level, ingested food is first processed in the brainstem and in higher cognitive regions at a later point in time. Besides distributing information about food intake to various regions in the brain, the NTS is responsible for the immediate control of automated mechanisms induced by food intake such as chewing, licking, and swallowing, and thus also for a number of reflex activities (Bradley, 2007). Higher cognitive regions such as reward-associated areas do not become active until later, e.g., during the evaluation of the food and reduction in FC 40 min after the meal might thus be based on a decoupling of early taste and later rewarding properties, such as are induced by satiety (Chen et al., 2016). Furthermore, there is evidence of functional connections of the NTS to the insular cortex, particularly during the process of swallowing (Daniels and Foundas, 1997). These connections might therefore be directly involved in the processing of food. Again, the insula is functionally connected to reward-related regions such as the putamen, caudate, pallidum and comprises the primary gustatory cortex with the frontal operculum. Our study therefore suggests that food intake changes FC patterns between the reward system and the gustatory system.

Regression analyses with oral fat sensitivity and FC patterns – in particular those between hypothalamic and limbic regions – showed dependencies on the fat content of a meal. We already demonstrated that the fat content of a meal affects hypothalamic activation changes differentially (Frank et al., 2012). It therefore goes to follow that in particular the FC network with the seed in the hypothalamus differs between the two yogurt conditions. Here, however, we particularly found the further interaction with oral fat sensitivity in a network that included hypothalamic, insular, amygdalar and hippocampal regions showing positive correlations of oral fat sensitivity with the change in FC for the high-fat condition only. The temporal cortex, most notably the insula, plays a crucial role in taste perception (Small et al., 2001; Small, 2010). Furthermore, the insular cortex receives afferents from the amygdala in the limbic cortex (Hoogeveen et al., 2015), which again is a major driver for conditioning processes and thus tightly linked to taste perception (Molero-Chamizo and Rivera-Urbina, 2017). Moreover, the amygdala is functionally connected to the hypothalamus during taste processes (Hoogeveen et al., 2015) and was shown to be particularly responsive to the perceptive intensity independent of valence (Small et al., 2003). With regard to the hippocampus, a number of hippocampal cells were recently shown to be particularly responsive to taste stimuli (Herzog et al., 2019). On the basis of our results, we thus demonstrate that individual oral fat perception modulates FC networks after the ingestion of a high- vs. a low-fat meal.

A different relation emerged for correlative patterns of FC between the DS with the somatosensory cortex. Here, oral fat sensitivity was negatively associated with FC for the low-fat condition, but not for the high-fat condition. Therefore, the higher the oral fat sensitivity, the more reduced the connectivity between the reward system and the somatosensory cortex after the intake of a low-fat yogurt. An earlier study showed the sensibility of the somatosensory cortex to the fattiness of samples placed on the tongue of the participants (Grabenhorst and Rolls, 2013). As suggested by our study, such effects might be associated with different FC to reward-related regions.

On the basis of the positive correlation of oral fat sensitivity between both days, an individual’s fat sensitivity is a relatively stable measure. Our reliability results of *r* = 0.66 were in line with previously reported reliability measures of oleic acid across separate study days (reported ICC = 0.64) (Newman and Keast, 2013). In addition, Newman et al. (2016) found no differences in fat taste thresholds due to a single high-fat meal immediately prior to threshold testing. The type of food ingested before investigating oral fat sensitivity should, however, be considered when interpreting imaging data. As our study showed, the association of oral fat sensitivity with neuronal connectivity patterns might depend on the fat-content of previously ingested food. A recent study showed distinct differential FC patterns after a high- vs. a low-fat meal, especially in patients suffering from functional dyspepsia (Lee et al., 2018). Changes in gastrointestinal functions might therefore also influence brain connectivity patterns in association with the fat content in a meal. Just as the study by Lee et al. (2018) provided interesting insight as to how the fat content of a meal influences the association between brain connectivity patterns and gastrointestinal functions, our study divulges additional information on the interaction of such functions with a focus on variabilities in taste perception.

One limitation that cannot be ignored is the small sample size which might cause the problem of underpowering. What is more, since we recruited males only to prevent cycle-related hormonal interactions, we cannot transfer our results to the female population. Our result interpretation is also limited to younger adults and normal-weight subjects. Furthermore, we are currently restricted to a limited FOV, which can hopefully soon be overcome by more advanced sequences.

## Conclusion

In summary, we have shown that oral fat sensitivity has to be observed in close association with the fat content of previously ingested meal when investigating underlying connectivity patterns. Oral fat sensitivity in particular determines FC patterns with the seed in homeostatic and reward regions, depending on the fat content of ingested food. Alternatively, any FC patterns influenced by the fat content of the ingested food might influence oral fat sensitivity. The study delivers important insight into the ongoing discussion of a potentially differential processing of low-fat meals and might be an underlying mechanism of previously shown associations of eating behavior with oral fat sensitivity.

## Data Availability

The datasets generated for this study are available on request to the corresponding author.

## Ethics Statement

All participants gave written informed consent prior to participation and the study was approved by the Ethics Committee of the University of Tübingen. The study was conducted in accordance with The Code of Ethics of the World Medical Association (Declaration of Helsinki) for experiments involving humans.

## Author Contributions

AF, HP, JH, KS, and SF-P designed the study. JMH, JM, RV, and SF-P collected the data. GC, JMH, JM, MR, RV, and SF-P analyzed the data. AF, HP, JH, and KS supervised the work. JMH, JM, MR, and SF-P drafted the manuscript. All authors revised the manuscript.

## Conflict of Interest Statement

The authors declare that the research was conducted in the absence of any commercial or financial relationships that could be construed as a potential conflict of interest.

## Footnotes

1 http://www.restfmri.net

2 http://www.fil.ion.ucl.ac.uk/spm

3 http://www.restfmri.net

4 https://www.psychometrica.de/correlation.html

## Abbreviations

DS: dorsal striatum
FC: functional connectivity
LH: lateral hypothalamus
NTS: nucleus tractus solitarii
VAS: visual analog scale
VMH: ventromedial hypothalamus.
