# Supplementary material for: Functional Connectivity Within the Gustatory Network Is Altered by Fat Content and Oral Fat Sensitivity – A Pilot Study
Source: Front Neurosci. 2019 Jul 10;13:725. doi: 10.3389/fnins.2019.00725 (PMC6636204; doi:10.3389/fnins.2019.00725)
Supplement: Supplementary file 1 [file Data_Sheet_1.PDF]

Supplementary Material:

Yogurt production:

Low- and high-fat yogurt was produced at the Institute of Food Science and Biotechnology (University of Hohenheim) similar to Frank et al. (1).

Caloric content and macronutrient composition are shown in Table S1. The large deformation test after 24 h of storage at 10 °C has been performed by means of rheological measurements (2) which revealed no difference between the low- and high-fat yogurt in terms of viscosity properties.

Table S1. *Macronutrient composition and caloric content of low- and high-fat yogurts.*

	C_F	C_P	C_L	*Energy value*
	wt%:wt%	*wt%:wt%*	*wt%:wt%*	*kcal/100 g*
Low-fat yogurt	0.11 (±0.004)	6.06 ± 0.06	4.61 (± 0.01)	39.06
High-fat yogurt	7.88 (± 0.005)	3.22 ± 0.02	4.51 (± 0.01)	97.33

CF, fat content; CL, lactose content; CP, protein content.
Mean ± SEM of 3 measurements (all such values).

Oral sensitivity testing:

Oleic acid (Sigma Aldrich, Germany), non-fat UHT milk (Weihenstephan, Molkerei Weihenstephan GmbH & Co. KG, Freising, Germany), gum arabic (Naturix24, Dransfeld, Germany), ethylenediaminetetraacetate (EDTA, VWR, Darmstadt, Germany), and paraffin oil (VWR, Darmstadt, Germany) were all food grade quality and purchased from commercial vendors.

Determination of detection thresholds for oleic acid were based on the ASTM E679 method (3), ISO 3972:2011 – Sensory analysis –Methodology –Method of investigating sensitivity of taste (4), and the protocols of Haryono et al. (5) and Giguere et al (6). A total of 8 concentrations were prepared (Table S2). A base solution had to be prepared according to Haryono et al. (5).

Here, long-life, non-fat UHT milk was mixed with 5% w/v gum Arabic (to emulsify and stabilize) and 0.01% w/v EDTA (to avoid oxidation) at 8000 rpm for 3 minutes (Ultra-Turrax T50, IKA ®-Werke-GmbH & Co. KG; Staufen, Germany). To prepare 8 fat concentrations), the appropriate amount of oleic acid, based on its density and molecular weight, was placed along with 5 % paraffin oil (to increase viscosity) in 8 beakers. Each beaker was filled up with base solution and homogenized for 30 seconds/100 mL (Polytron ® System PT 2500 E, Kinematica AG, Luzern, Switzerland). Control samples were prepared in the same way but without adding oleic acid.

Table S2. Fatty concentrations.

oral fat sensitivity index	Oleic acid (mM)	Taster-status
9	0.04	taster
8	0.12	taster
7	0.37	taster
6	1.10	taster
5	3.30	taster
4	9.90	taster
3	29.70	taster
2	89.10	taster
1	267.30	nontaster

Concentration steps 9-2 were the actual concentrations of oleic acid. Concentration step 1 is an extrapolation of the last concentration, which is used for nontasters (who did not identify the highest concentration on offer).

A triangle forced choice method was used to determine detection thresholds. To avoid olfactory and visual cues, participants were instructed to wear nose clips and red glasses during testing. Participants received a set of 8 rows with sets of 3 samples in ascending order with the bottom row containing the lowest concentration. Samples contained 10 mL each and were labelled with a 3-digit number. Each set contained one test sample with the stimulus and two control samples in randomized order. Participants were instructed to taste each sample once, starting from the left to the right, retain it in the mouth for 5 seconds before expectorating it. After having tasted all 3 samples of a set, participants were asked to “identify the odd one out” (detection threshold). When no difference between the samples could be perceived, participants were asked to choose

a sample (forced choice). Between sample sets, participants were briefed to neutralize their mouth with water, bread (matzo) and to use a tongue scraper to minimize a remaining fat film in the oral cavity.

If the participant was not able to identify the highest concentration offered (nontaster), a value beyond test series was extrapolated. Subsequently, a nontaster was assigned an extrapolated concentration step with the value 1.

Gastric volume over the course of measurements

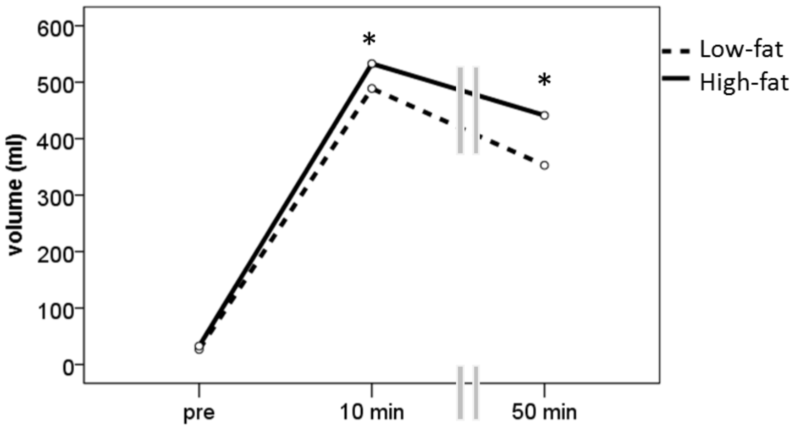

Figure S1: Gastric volume measured to three time points.

References

1. Frank S, Linder K, Kullmann S, Heni M, Ketterer C, Cavusoglu M, Krzeminski A, Fritsche A, Haring HU, Preissl H, et al. Fat intake modulates cerebral blood flow in homeostatic and gustatory brain areas in humans. Am J Clin Nutr 2012;95(6):1342-9. doi: 10.3945/ajcn.111.031492.
2. Krzeminski A, Großhable K, Hinrichs J. Structural properties of stirred yoghurt as influenced by whey proteins. LWT - Food Science and Technology 2011;44(10):1298-311.
3. ASTM. American Society for Testing and Materials (ASTM) E679-04. Standard practice for determination of odor and taste thresholds by a forced choice ascending concentration series method of limits. 2011.
4. ISO. International Standard Organisation (ISO) 3972. Sensory analysis - methodology - method of investigating the sensitivity of taste. 2011.
5. Haryono RY, Sprajcer MA, Keast RS. Measuring oral fatty acid thresholds, fat perception, fatty food liking, and papillae density in humans. J Vis Exp 2014(88). doi: 10.3791/51236.
6. Giguere JF, Piovesana Pde M, Proulx-Belhumeur A, Dore M, Sampaio Kde L, Gallani MC. Reliability of a Simple Method for Determining Salt Taste Detection and Recognition Thresholds. Chem Senses 2016;41(3):205-10. doi: 10.1093/chemse/bjv080.
